# The prevalence and predictors of previous prostate cancer screening among men attending primary healthcare centers in Riyadh, Saudi Arabia

**DOI:** 10.1080/07853890.2026.2628366

**Published:** 2026-03-14

**Authors:** Saad Alshahrani, Mamdouh M. Shubair, Atheer Abdulrahman Alfallaj, Badr F. Al-Khateeb, Awad Alshahrani, Nura Almutairi, Lubna Alnaim, Khadijah Angawi, Amani Alharthy, Fatmah Othman, Ashraf El-Metwally

**Affiliations:** a Department of Surgery, Division of Urology, College of Medicine, Prince Sattam Bin Abdulaziz University, Alkharj, Saudi Arabia; b School of Health Sciences, University of Northern British Columbia (UNBC), Prince George, Canada; c Medical Imaging Department, King Saud Medical City, Riyadh First Health Cluster, Riyadh, Saudi Arabia; d King Abdullah International Medical Research Center, Riyadh, Saudi Arabia; e College of Medicine, King Saud bin Abdulaziz University for Health Sciences, Riyadh, Saudi Arabia; f Ministry of the National Guard-Health affairs, Riyadh, Saudi Arabia; g Ministry of Health, Riyadh Second Health Cluster, Riyadh, Saudi Arabia; h Clinical Nutrition Department, College of Applied Medical Sciences, King Saud Bin Abdulaziz University for Health Sciences, Al Ahsa, Saudi Arabia; i Department of Health Services and Hospital Administration, Faculty of Economics and Administration, King Abdulaziz University, Jeddah, Saudi Arabia; j Department of Health Science, College of Health and Rehabilitation Sciences, Princess Nora Bint Abdulrahman University, Riyadh, Saudi Arabia; k College of Public Health and Health Informatics, King Saud Bin Abdulaziz University for Health Sciences, Riyadh, Saudi Arabia

**Keywords:** Predictors, primary healthcare, prostate cancer, screening, Saudi Arabia

## Abstract

**Background:**

Early detection through screening is crucial for improving treatment outcomes and reducing disease burden. . This study investigates the predictors of previous prostate cancer screening among Saudi men attending primary healthcare centers (PHCs) in Riyadh, Saudi Arabia.

**Methods:**

A cross-sectional study was conducted from March to July 2023, involving 6,177 men attending 48 PHCs in Riyadh, selected *via* multistage cluster sampling. Unadjusted and adjusted logistic regression analyses were performed to identify significant predictors of screening, with statistical significance set at *p* < 0.05.

**Results:**

A total of 6,177 men participated in the study. Age distribution was 31% under 50, 48.4% aged 50–75, 20.5% 75+ years . Only 1.5% of participants reported having undergone previous prostate cancer screening. crude proportions of screening were 0.9%, 1.7%, and 1.3% for the <50, 50 − 75, and ≥75 age groups, respectively. In adjusted analysis, age 50–75 years (AOR: 3.07, 95% CI: 1.73–5.46), unemployment (AOR: 3.69, 95% CI: 2.33–5.85), health insurance coverage (AOR: 2.98, 95% CI: 1.93–4.61), smoking (AOR: 4.52, 95% CI: 2.73–7.49), and history of heart disease (AOR: 3.04, 95% CI: 1.44–6.42) were significant predictors of previous prostate cancer screening.

**Conclusion:**

The extremely low prevalence of previous prostate cancer screening in this population underscores urgent need to improve access to PSA testing. Employment status, insurance coverage, smoking, and history of heart disease were significant predictors of screening uptake. These findings highlight need for both targeted and general interventions and support development of a national strategy for PSA testing among asymptomatic men in Saudi Arabia.

## Introduction

Prostate cancer stands as a significant global health concern, representing a leading cause of cancer-related morbidity and mortality among men [1]. In 2020, an estimated 1.4 million new cases and over 375,000 deaths were reported worldwide, with incidence rates highest in Australia/New Zealand, North America, and Northern Europe, and mortality rates highest in sub-Saharan Africa and Latin America/Caribbean [2,3]. While mortality from prostate cancer has generally declined in many high-income countries such as the United States and Western Europe, largely due to widespread PSA testing and improvements in treatment, incidence and mortality rates continue to rise in several regions of Africa, Asia, and Latin America [2,3]. Data from the Saudi Cancer Registry demonstrate that prostate cancer incidence remains relatively low in Saudi Arabia, ranging from 0.2 to 1.4 per 100,000 between 2008 and 2017 [4]. This is considerably lower than the incidence observed in many European countries, such as Bulgaria (147.7 100,000), Czechia (132.9 100,000), and Hungary (135.6 per 100,000 100,000), and North America, where rates reach approximately 110 per 100,000 in several regions [2,3]. Mortality rates in Saudi Arabia are also relatively low compared with high-burden regions like Sub-Saharan Africa and parts of the Caribbean (e.g. Barbados 40.3 per 100,000) [2,3]. Consequently, organized, population-based screening in Saudi Arabia may be less cost-effective than in countries such as the Czech Republic, Lithuania, and Sweden, which implement organized, population-based national screening programs for prostate cancer.

Evidence from regional studies indicates that most Saudi men are diagnosed with prostate cancer at an advanced age and with elevated PSA levels. For example, in Aseer, 96% of men diagnosed were older than 60 years, with 92% presenting with PSA >4 ng/ml [5]. Similarly, studies from Madinah reported a mean age of 70.9 years for prostate cancer patients, with most cancers moderately differentiated (Gleason score 6–7) and PSA levels often exceeding 100 ng/ml [6]. In Jeddah, incidental prostate cancer was detected in 15% of presumed benign cases, with 92.8% of cancers having Gleason score ≥6 [7]. These findings reflect advanced age and high PSA at diagnosis rather than Gleason score as an indicator of late presentation.

Early detection through screening plays a pivotal role in improving treatment outcomes and reducing the burden of this disease [8]. While established screening guidelines exist in many parts of the world [9,10], their uptake and effectiveness can vary considerably across different populations and healthcare settings [11–13]. Understanding the factors that influence men’s decisions to undergo previous prostate cancer testing is crucial for developing targeted interventions to promote early diagnosis and improve public health.

In Saudi Arabia, the healthcare system is primarily government-funded, with primary healthcare centers (PHCs) serving as the first point of contact for most men [14]. PHCs provide preventive services, health education, and routine screenings, with referrals to specialized care in hospitals when necessary [15]. This system ensures broad access to basic healthcare services; however, the organization and utilization patterns may influence prostate cancer screening uptake. Currently, Saudi Arabia has no official national guidelines recommending PSA testing for asymptomatic men, highlighting the importance of understanding testing practices in the primary healthcare context.

Within the Kingdom of Saudi Arabia, the landscape of cancer care is evolving rapidly alongside its healthcare infrastructure [16]. As the population ages and awareness of men’s health issues grows, understanding the specific context of prostate cancer screening becomes increasingly important [8]. The predictors of prostate cancer screening adherence within this primary healthcare context in Saudi Arabia remain underexplored, and available studies often report late-stage diagnoses and elevated PSA at presentation.

Existing literature on prostate cancer screening often focuses on Western populations and healthcare systems [17–20]. While these studies provide valuable insights into factors such as age, family history, socioeconomic status, and health beliefs [18–20], their direct applicability to the Saudi Arabian context may be limited due to cultural, societal, and healthcare system differences. For instance, cultural norms surrounding health-seeking behavior, the role of family in health decisions, and the specific organization of primary healthcare services in Saudi Arabia could significantly influence screening uptake [15,21]. Therefore, a clear gap exists in the literature regarding the specific predictors of previous prostate cancer screening among Saudi men utilizing primary healthcare services within Riyadh.

This research endeavors to address this gap by investigating the factors that predict previous prostate cancer screening among Saudi men attending primary healthcare settings in Riyadh. Understanding these predictors is essential for several reasons. Firstly, it will provide empirical evidence specific to the Saudi context, allowing for the development of culturally tailored interventions to increase screening rates. Secondly, it will contribute to a more nuanced understanding of men’s health behaviors within the region, potentially informing broader public health strategies.

## Methods

### Study design and setting

This study was a cross-sectional survey conducted between March and July 2023 in Riyadh, Saudi Arabia. The primary objectives were to assess the prevalence of prostate cancer screening among men and to identify sociodemographic, behavioral, and clinical factors influencing screening behavior. The present study represents a sub-analysis of a larger needs-assessment dataset conducted in Riyadh, Saudi Arabia [22–25], with a specific focus on prostate cancer.

### Participants’ eligibility

The study included adult men aged 18 years and older, attending PHCs, irrespective of residency status. While men under 50 are generally not recommended for routine prostate cancer screening, they were included to allow estimation of baseline screening prevalence and identification of early predictors. Exclusion criteria included healthcare professionals and individuals who declined informed consent. The exclusion of women, younger men, and healthcare professionals ensured that the study population appropriately reflected men who are potential candidates for prostate cancer screening in primary healthcare settings. This approach allows for a representative sample of the target population while maintaining the focus on the relevant study group. The final analytical sample included 6,177 men who completed the survey.

### Sampling strategy

A multi-stage cluster sampling method was implemented to obtain a representative sample of men attending primary healthcare centers (PHCs) in Riyadh. Riyadh is divided into three health clusters, each encompassing multiple primary, secondary, and tertiary healthcare facilities. Cluster 2, comprising 105 PHCs, 7 hospitals, and 7 tertiary care facilities and serving approximately 3.7 million residents, was included as part of an ongoing needs assessment activity across all clusters to evaluate population health needs and plan interventions. Random selection of 48 PHCs within Cluster 2 was performed using a stratified random sampling technique. Within each selected PHC, systematic random sampling of attendees was applied: data collectors approached every fourth adult male in the waiting area and invited him to participate in the study. Participation was voluntary, and informed consent was obtained prior to the survey.

### Questionnaire development and content

The survey instrument, developed by the Central Health Services Reform Management Team and validated in Hail City, was used across all health clusters in Saudi Arabia. It comprised items on self-reported health status, medical history (e.g. heart disease, diabetes, hypertension, obesity, hypercholesterolemia), health behaviors (e.g. smoking, fast food consumption, physical activity, vitamin use), and sociodemographic characteristics (e.g. age, education, employment, marital status, insurance coverage). Smoking was assessed based on self-report, using a single question asking whether the participant currently smokes any tobacco products (yes/no). Participants were also asked about prostate cancer screening: ‘Have you ever undergone prostate cancer screening, using at least digital rectal examination method?’ (Response options: Yes/No). The full questionnaire is provided in Supplementary File 1.

### Validity and reliability of the questionnaire

A strict process was put in place to make sure the questionnaire was valid and reliable. A panel of 15 experts, including healthcare doctors and public health professionals, looked at the questions to see if they were relevant, accurate, and suitable. This proved that the content was valid. Based on their suggestions, several questions in the questionnaire were changed or taken out to make the data collecting instrument work better. A pilot study with 200 participants was used to find out if the questions were clear, hard, and easy to understand. Trained data collectors read the questions out loud during interviews to help people comprehend them better. A test-retest method was used to check how reliable the instrument was. In this method, 100 people from the pilot study filled out the questionnaire again over the phone after certain changes were made. The test-retest reliability value of 0.83 showed that the results were quite consistent and reliable. Furthermore, to ensure linguistic accuracy, the questionnaire was subjected to a rigorous translation procedure from English to Arabic and subsequently back to English, thereby validating the precision and integrity of the instrument in both languages. The preliminary testing of the questionnaire was conducted in Hail City instead of Riyadh, following a strategic decision by the Central Health Services Reform Management Team.

### Data collection procedures

Data were collected using interviewer-administered electronic surveys on iPads or Android tablets, conducted face-to-face. Data collectors read questions aloud and entered responses directly into the electronic system. Data collection at each PHC lasted approximately 8 h/days. Participation was voluntary, and written informed consent was obtained electronically on the tablet prior to survey administration. Participants were not reimbursed for their time. All responses were anonymous and confidential.

### Statistical analysis

We reported descriptive statistics, followed by conducting regression analysis. For categorical variables, such as education level, employment, marital status, general health perception, and insurance coverage, frequencies and percentages were used to explain the sample’s characteristics. The age variable was also divided into distinct groups to look at how participants were spread out across different age groups. For the unadjusted analysis, univariate logistic regression was utilized to investigate the relationship between each independent variable and the binary outcome variable (previous prostate cancer screening: Yes/No). Variables were first assessed in univariate logistic regression analyses. A *p* value threshold of <0.25 was used to identify variables eligible for inclusion in the multivariable logistic regression model, a commonly recommended criterion to avoid excluding potentially important predictors at the preliminary stage. Variables meeting these criteria, such as age, employment status, insurance coverage, smoking status, and history of heart disease, were entered simultaneously into the multivariable model. Variables with *p* values ≥0.25 in the univariate analysis (e.g. education and marital status) were excluded. The final selection of variables therefore relied on a single, consistent statistical criterion (*p* < 0.25) complemented by clinical relevance. Adjusted odds ratios (AORs) with 95% confidence intervals (CIs) were reported, and *p* < 0.05 was considered statistically significant. All analyses were performed using SPSS version 26.

### Study results

#### Participant selection

A total of 16,752 individuals were approached to participate in the electronic survey, of whom 14,239 (85%) completed it. Of these, 6,177 men met the eligibility criteria and were included in the final analysis.

#### Prostate cancer screening uptake

Across the sample of 6,177 men, 1.5% reported ever undergoing PSA testing. Screening prevalence varied by age group. Men younger than 50 years reported uptake of 0.9%, those aged 50–75 years reported 1.7%, and men aged 75 years or older reported 1.3%.

#### Sociodemographic and behavioral characteristics

Table 1 presents the sociodemographic and behavioral characteristics of the 6,177 male participants. Most men were aged 50 years or older, with nearly half between 50 and 75 years. The majority were married and had attained at least high school education. Two-thirds were employed, but fewer than one-quarter reported having health insurance coverage.

**Table 1.** Sociodemographic and behavioral characteristics of Saudi men attending primary healthcare centers in Riyadh, March–July 2023 (*n* = 6,177).

Characteristics	*n*	%
**Sociodemographic characteristics**		
**Age groups**		
<50 years	1,917	31.0
50–75 years	2,991	48.4
At least 75 years	1,269	20.5
**Married**	3,961	64.1
**Education level**		
Primary	232	3.8
Up to High School	1,778	28.8
College/University	3,109	50.3
Others	1,058	17.1
**Employed**	4,178	67.6
**Insurance coverage**	1,534	24.8
***Behavioral and clinical characteristics***		
**Vitamin use**	4,174	67.6
**Fast food consumption**	4,594	74.4
**Physical active**	3,935	63.7
**Smoking history**	1,981	32.1
**Hypertension**	671	10.9
**Heart disease**	191	3.1
**Obesity**	265	4.3
**Diabetes**	848	13.7
**Screening for prostate cancer**	86	1.5

In terms of health behaviors, fast food consumption and vitamin use were common, while about one-third of participants reported a history of smoking. Hypertension and diabetes were among the most frequently reported comorbidities.

#### Sociodemographic determinants for prostate cancer screening

Table 2 presents the sociodemographic determinants of previous prostate cancer screening among the 6,177 participants. Individuals aged 50 to 75 years had three-fold higher odds of screening compared with those younger than 50 years (AOR 3.07, 95% CI 1.73–5.46). Unemployed participants showed nearly four-fold higher odds of screening than employed men (AOR 3.69, 95% CI 2.33–5.85). Those with health insurance had approximately three-fold higher odds of screening compared with uninsured participants (AOR 2.98, 95% CI 1.93–4.61).

**Table 2.** Sociodemographic determinants of previous prostate cancer screening among Saudi men attending primary healthcare centers in Riyadh, March–July 2023.

Predictors	Unadjusted analysis		Adjusted analysis	
	OR (95% CI)	*p* value	AOR (95% CI)	*p* value
**Age**				
<50 years	Ref	0.049	Ref	
50–75 years	1.98 (1.14–3.43)		3.07 (1.73–5.46)	<0.001
At least 75 years	1.52 (0.77–2.98)		1.58 (0.80–3.12)	
**Education**				
Primary	Ref		NA	
Up to High School	2.49 (0.33–18.73)	0.374		
College/University	4.31 (0.59–31.30)			
Others	1.98 (0.25–15.72)			
**Marital status**			NA	
Single	Ref			
Married	1.45 (0.90–2.33)	0.12		
**Employment status**				
Employed	Ref	<0.001	1.00 (Ref)	<0.001
Unemployed	2.32 (1.52–3.56)		3.69 (2.33–5.85)	
**Insurance coverage**				
No	Ref	<0.001	1.00 (Ref)	<0.001
Yes	2.55 (1.66–3.92)		2.98 (1.93–4.61)	

OR: Odds ratio; AOR: Adjusted odds ratio; 95% CI: 95% confidence interval; LL: Lower limit; UL: Upper limit; NA: Not applicable because education and marital status were not statistically significant in the univariate model.

Multivariable analysis: AORs are estimated after adjusting for variables significant in the univariate analysis at *p* < 0.10, including age, employment status, and insurance coverage.

***Behavioral and health-related determinants of prostate cancer screening***

Table 3 shows behavioral and health-related determinants. In unadjusted analyses, smoking, physical activity, vitamin supplement use, obesity, and heart disease were associated with screening uptake, while diabetes and fast-food consumption were not. Smoking (OR 5.62, 95% CI 3.44–9.02), physical activity (OR 3.24, 95% CI 1.79–5.86), vitamin use (OR 2.99, 95% CI 1.62–5.52), obesity (OR 2.33, 95% CI 1.11–4.87), and heart disease (OR 5.35, 95% CI 2.86–10.03) showed the strongest associations.

**Table 3.** Behavioral and health-related determinants of previous prostate cancer screening among Saudi men attending primary healthcare centers in Riyadh, March–July 2023.

Determinants	Unadjusted analysis		Adjusted analysis	
	OR (95% CI)	*p* value	AOR (95% CI)	*p* value
**Smoking**				
No	Ref	<0.001	Ref	<0.001
Yes	5.62 (3.44–9.02)		4.52 (2.73–7.49)	
**Physical activity**				
No	Ref	<0.001	Ref	0.086
Yes	3.24 (1.79–5.86)		1.83 (0.92–3.66)	
**Vitamin Use**				
No	Ref	<0.001	Ref	0.067
Yes	2.99 (1.62–5.52)		1.86 (0.96–3.62)	
**Fast food consumption**				
No	Ref	0.083	Ref	0.237
Yes	1.64 (0.94–2.87)		0.67 (0.35–1.30)	
**Diabetes**				
No	Ref	0.80	NA	
Yes	0.92 (0.49–1.74)			
**Hypertension**				
No	Ref	0.02	Ref	0.234
Yes	1.90 (1.09–3.28)		1.47 (0.78–2.78)	
**Obesity**				
No	Ref	0.025	Ref	0.966
Yes	2.33 (1.11–4.87)		1.02 (0.44–2.35)	
**Heart disease**				
No	Ref	<0.001	Ref	0.003
Yes	5.35 (2.86–10.03)		3.04 (1.44–6.42)	

OR: Odds ratio; AOR: Adjusted odds ratio; 95% CI: 95% confidence interval; LL: Lower limit; UL: Upper limit; NA: Not applicable because diabetes mellitus history was not statistically significant in the univariate model (*p* = 0.79).

All other variables were statistically significant at *p* < 0.10 in the univariate model.

Multivariable analysis: AORs are estimated after mutually adjusting for sociodemographic variables significant in Table 2 (age, employment, and insurance coverage), as well as all behavioral factors significant in the univariate analysis at *p* < 0.10, except for diabetes mellitus history.

In the multivariable analysis, smoking and heart disease remained independent predictors. Smokers had over four-fold higher odds of screening (AOR 4.52, 95% CI 2.73–7.49), and individuals with heart disease had threefold higher odds (AOR 3.04, 95% CI 1.44–6.42). Physical activity (AOR 1.83, 95% CI 0.92–3.66) and vitamin use (AOR 1.86, 95% CI 0.96–3.62) showed borderline associations but were not statistically significant. Fast-food consumption, obesity, and hypertension were not associated with screening in adjusted analyses.

## Discussion

In our study, conducted among individuals utilizing primary healthcare services in Riyadh, the prevalence of reported prostate cancer screening was 1.5%. This rate is considerably lower than those reported in Western countries [26,27], indicating a potential lack of awareness, access barriers, or cultural factors that impede screening uptake in Saudia Arabia [28]. Population-based screening programs for prostate cancer exist in only a few countries, specifically the Czech Republic, Lithuania, and Sweden, and routine PSA testing is not mandated in any country. Although basic healthcare services are widely accessible through the public sector in Saudi Arabia, opportunistic screening remains dependent on individual physician recommendations or patient-initiated requests, which may limit uptake among individuals with low health literacy or limited awareness. Additionally, cultural perceptions surrounding urological symptoms and reluctance to discuss male reproductive health may further reduce screening engagement. Given the absence of national recommendations and the relatively low prostate cancer mortality in Saudi Arabia, system-level strategies should be carefully evaluated to ensure that resource allocation aligns with national priorities; broad public screening campaigns or routine counselling might not represent the most efficient use of primary care capacity.

The current study results reported that age emerged as a strong predictor, with men aged 50–75 years being over three times more likely to undergo screening than those younger than 50 years. This positive association aligns with international recommendations that typically consider this age range appropriate for screening among men at average risk [29–31]. These findings indicate that screening uptake was highest in the 50–75-year group, consistent with expected age-related increases in healthcare engagement and guideline-driven physician recommendations [31,32].

Employment status also showed a significant association, with unemployed individuals nearly four times more likely to report previous screening than employed individuals. This unexpected pattern may reflect greater time availability among unemployed individuals or more frequent use of publicly funded services. In contrast, employed individuals may experience competing work constraints or variable insurance coverage. While studies elsewhere often show higher screening rates among employed individuals [29,30], context-specific factors within Saudi Arabia, such as insurance structures and appointment flexibility, may influence screening differently.

Insurance coverage was another important determinant; insured participants were almost three times more likely to be screened compared to uninsured individuals. This aligns with international evidence linking insurance with greater use of preventive services [29,31,33,34]. Because insurance reduces out-of-pocket costs for physician visits and PSA testing, individuals insured experience fewer financial barriers. In Saudi Arabia, the coexistence of public and private healthcare systems may contribute to disparities: uninsured expatriates and some unemployed citizens may lack consistent access to preventive services. These findings collectively reinforce the importance of financial protection reforms to support equitable access to cancer prevention.

Interestingly, smoking also demonstrated a strong positive association with screening, with smokers being over four times more likely to undergo prostate cancer screening. Smoking is known to be associated with multiple chronic diseases [35,36], and possibly aggressive forms of prostate cancer [37,38]. Individuals who smoke, particularly former smokers, may have more frequent medical encounters or higher health risk awareness, increasing opportunities for screening discussions. Previous studies consistently show higher screening rates among former smokers across several cancer types [39–41], likely due to lifestyle changes following cessation [41,42]. The lack of differentiation between current and former smokers in our data limits interpretation, but the overall association suggests the healthcare system may be leveraging clinical encounters to promote preventive care.

The history of heart disease was similarly associated with a threefold increase in screening uptake. This likely reflects increased healthcare utilization among individuals with chronic disease [43,44], providing more opportunities for physicians to recommend age-appropriate preventive screenings. Managing one chronic condition may also elevate general health awareness, leading to greater participation in other preventive practices [45].

Obesity showed no significant association with screening in this study. Despite its established link to various cancers, including aggressive prostate cancer, obesity did not predict screening behavior. This may suggest that although obese individuals frequently interact with the healthcare system, these encounters focus on chronic disease management rather than preventive cancer screening. Other factors—including health literacy, stigma, and psychosocial barriers—may also contribute to the lack of association.

Other variables, including marital status, education, income, diabetes, hypertension, and fast-food consumption, did not remain significant in adjusted models. These findings indicate that structural factors such as age, insurance status, and employment may exert stronger influence over screening behavior than individual lifestyle or demographic characteristics. Individuals managing chronic conditions may prioritize disease management, leaving limited attention for preventive cancer screening; alternatively, competing priorities or residual confounding may obscure associations. Recognizing these null findings is important, as they reinforce the need to strengthen system-level facilitators rather than focusing solely on individual behavioral change.

## Strengths and limitations

This study benefits from several strengths. The use of a multi-stage cluster sampling method from a large and diverse population within the Riyadh region enhances the generalizability of the findings to individuals utilizing primary healthcare services in this major urban center of Saudi Arabia. The large sample size provides adequate statistical power to detect significant associations. Furthermore, the comprehensive questionnaire, developed as part of a national health reform initiative, allowed for the examination of a wide range of potential predictors.

However, some limitations should be considered when interpreting these findings. The cross-sectional design limits the ability to establish temporal relationships or causality between the identified predictors and screening uptake, meaning the observed associations cannot confirm directionality. The reliance on self-reported screening behavior may have introduced recall or social desirability bias, potentially leading to either overestimation or underestimation of true screening rates; to mitigate this, trained data collectors administered the survey in a standardized manner to improve accuracy. While the sample was drawn from individuals attending primary healthcare centers, the main entry point to care for most adult residents, men who do not visit PHCs may differ in health-seeking behavior, comorbidity burden, or awareness, possibly affecting the generalizability of our findings. Unmeasured factors, such as family history of cancer, frequency of physician counseling, or prior exposure to health education campaigns, may have influenced screening behavior; future studies should aim to capture these variables to refine our understanding. Finally, as data were collected only from PHCs in Riyadh, the findings may not fully represent men utilizing private or tertiary healthcare facilities or those living in other regions of the country. Despite these limitations, the study provides valuable insights into the structural and behavioral determinants of prostate cancer screening in this population and highlights areas for targeted interventions.

## Conclusion and implications for Saudi Arabia

The extremely low prevalence of previous prostate cancer screening in this population underscores the urgent need to improve access to PSA testing. Prostate cancer screening behavior in Saudi Arabia is driven more by structural enablers, such as access to insurance, stable employment, and routine engagement with healthcare due to age or comorbid conditions, than by individual lifestyle or educational factors. These results suggest that improving screening uptake will require system-level strategies, including expanding insurance coverage, integrating screening into routine chronic disease care, and addressing barriers for unemployed and uninsured populations. Both targeted interventions for high-risk groups and general strategies to increase awareness and access are warranted. Collectively, our study points to the need for a national strategy for PSA testing of asymptomatic men to ensure equitable early detection of prostate cancer in Saudi Arabia.

## Supplementary Material

English Questionnaire.pdf

IANN-2025-3933.R3 supp file legends.docx

## Data Availability

The dataset(s) supporting the conclusions of this article will be made available by the corresponding author upon reasonable request.
