# Supplementary material for: The prevalence and predictors of previous prostate cancer screening among men attending primary healthcare centers in Riyadh, Saudi Arabia
Source: Ann Med. 2026 Mar 14;58(1):2628366. doi: 10.1080/07853890.2026.2628366 (PMC12990262; doi:10.1080/07853890.2026.2628366)
Supplement: English Questionnaire.pdf [file IANN_A_2628366_SM3668.pdf]

HEALTH NEED ASSESSMENT QUESTIONNAIRE

1. Name:
   ……………………….

2. YOUR sex:
☐ Female
☐ Male

3. YOUR marital status:
☐ Single/Not married
☐ Married
☐ Divorced
☐ Widowed

4. YOUR current age:
………………………………

5. YOUR highest level of education completed (check one):
☐ Illiterate school
☐ Primary
☐ Did not finish high school
☐ High school graduate
☐ Some college
☐ College graduate
☐ Other ……………………….

6. How many people currently live in YOUR household?
☐ 1-2
☐ 3-5
☐ 6 or more

7. How many adults aged 65 or older currently live in your household?
☐ 0
☐ 1-2
☐ 3-5
☐ 6 or more

8. How many children under 18 years of age live in your household?
☐ 0
☐ 1-2
☐ 3-5
☐ 6 or more

9. Do you rent or own where you live?
☐ Rent
☐ Own
☐ Other ……………………

………

10. What is YOUR employment status? (check all that apply)
☐ Employed full-time
☐ Employed part-time
☐ Full-time student
☐ Part-time student
☐ Part-time student
☐ Full-time homemaker
☐ Retired
☐ Unemployed
☐ Unemployed more than one year
☐ Unemployed less than one year
☐ Unemployed due to disability or illness

11. What is your annual household income?
…………………………..

12. What kinds of insurance do YOU (and/or your family) have currently?
☐ Health
☐ Dental
☐ Vision
☐ Do not have insurance

13. What type of health insurance do YOU (and/or your family) have currently?
☐ Self-insured
☐ Employer-provided
☐ Other………...
☐ Do not know
☐ Do not have health insurance

14. If everyone living in your household does not have insurance, who is NOT currently covered? (check all that apply)
☐ Entire family not covered
☐ At least one adult
☐ All adults
☐ Child(ren) 6 or older
☐ Child(ren) less than age 6
☐ Do not know

15. What is YOUR current health status?
☐ Poor
☐ Fair
☐ Good
☐ Very good
☐ Excellent

16. When was YOUR last routine doctor's visit?
☐ Within last 12 months
☐ Within last 13-18 months
☐ Within last 19-24 months
☐ Between 2 and 5 years
☐ Over 5 years ago
☐ Never had routine visit

17. How many days have YOU been too sick to work or carry out your usual activities during the past 30 days?
☐ None
☐ 1-2 days
☐ 3-5 days
☐ 6-10 days
☐ More than 10 days

18. When was YOUR last routine dentist visit to get a checkup (not for an emergency)?
☐ Within the last year
☐ 1-2 years ago
☐ 3-5 years ago
☐ 5 or more years ago
☐ I have never been to a dentist for a checkup

19. Have YOU ever had health issues due to any of the following? (check all that apply)
☐ Alcohol abuse
☐ Lack of pregnancy care
☐ Stress
☐ Drug abuse/addiction
☐ Family violence
☐ None of the above

20. When/If you were ever pregnant, did you receive prenatal care?
☐ Yes
☐ No
☐ Not sure
☐ Does not apply

21. When/If you ever gave birth, did you breastfeed?
☐ Yes
☐ No
☐ Not sure
☐ Does not apply

22. Where do YOU go for routine health care? (check all that apply)
☐ Physician's Office
☐ Hospital Emergency Room
☐ Health Department Clinic
☐ Urgent Care Center
☐ Chiropractor
☐ PHC
☐ Eye doctor
☐ Dentist
☐ Others
☐ Do not seek health care

23. How often are YOU able to visit a doctor when needed?
☐ Always
☐ Sometimes
☐ Seldom
☐ Never

24.If you answered seldom or never to #23, choose why were not always able to visit a doctor when needed:
☐ No insurance
☐ Too expensive/cannot afford
☐ Couldn't get appointment
☐ Lack of transportation
☐ Doctors is too far away
☐ Other

25.Select any of the following preventive procedures YOU have had in the last year (check all that apply):
☐ Mammogram
☐ Pap smear
☐ Glaucoma test
☐ Flu shot
☐ Colon/rectal examination
☐ Blood pressure check
☐ Blood sugar check
☐ Skin cancer screening
☐ Prostate cancer digital screen
☐ Prostate cancer PSA blood screen
☐ Cholesterol screen
☐ STI (sexually Transmitted Infections) screening
☐ Vision screening
☐ Hearing screening
☐ Cardiovascular screening
☐ Bone density test
☐ Dental exam

26. Are the children currently living in your home current on their immunizations?
☐ yes
☐ No
☐ Do not know
☐ Does not apply

27. Where do the children currently living in your home go for routine health care? (check all that apply)
☐ Physician's Office
☐ Hospital Emergency Room
☐ Health Department Clinic
☐ Urgent Care
☐ Chiropractor
☐ Community Clinic
☐ Eye Doctor
☐ Dentist
☐ Other
☐ Not applicable

28. Check all of the following that describe your home or your household?	29. What sources do you use to obtain most health-related information? (check all that apply)	31. How often do you travel outside of your Region for medical care?	34. If you seek medical care in the private sector, why?
☐ Smoke detector<br>☐ Carbon monoxide detector<br>☐ Primary source of drinking and cooking water is a private well or Cistern<br>☐ Food is put back into the refrigerator within the 2 hours after a meal<br>☐ Trash removed at least weekly<br>☐ Septic tank<br>☐ Pet (dog, cat, reptile)<br>☐ Family fire safety plan /evacuation plan<br>☐ Children know how to dial 999 for emergencies<br>☐ Children know their phone number and address<br>☐ Have one or more fire extinguishers<br>☐ Have one or more types of aerobic exercise equipment<br>☐ Internet access<br>☐ Provide care for an older adult	Friends or family<br>☐ Doctor/nurse/pharmacists<br>☐ Newspaper/magazine/TV<br>☐ Health Department<br>☐ Mosques<br>☐ School<br>☐ Internet<br>☐ Social Media<br>☐ Other ……………………..<br><br>30.What person or place do you feel is MOST responsible for providing you with health information (check only one):<br>☐ Mosques/Faith<br>☐ Doctor/nurses<br>☐ Hospital<br>☐ Health Department<br>☐ Yourself<br>☐ Employer<br>☐ Internet<br>☐ TV<br>☐ MoH<br>☐ Community<br>☐ Other: …………………….	☐ Always<br>☐ Sometimes<br>☐ Seldom<br>☐ Never<br><br>32. If you travel outside of your Region for medical care, what services do you seek? (check all that apply)<br>☐ Medical/doctor appointments<br>☐ Outpatient treatment<br>☐ Hospitalization<br>☐ Dental appointments<br>☐ Laboratory order tests<br>☐ X-rays<br>☐ Vision appointments<br>☐ Other …………………..<br><br>33. How often you seek medical care in private sector?<br>☐ Always<br>☐ Sometimes<br>☐ Seldom<br>☐ Never	☐ Services not available in public sector<br>☐ Better quality<br>☐ I have insurance<br>☐ Closer to work/home<br>☐ Too hard to get appointment with public doctors<br>☐ Other ……………………<br><br>35. If you travel outside of your county for medical care, why?<br>☐ Services not available in my county<br>☐ Better quality elsewhere<br>☐ Recently moved to this county<br>☐ Too hard to get appointment with local doctor<br>☐ Other ………………………<br>☐ N/A (do not travel outside of my county for medical care

36. In the following section, how often do YOU do the following?
(*N/A= does not apply to you)

	Almost Always	Sometimes	Never	N/A*
a.Wear a seat belt	☐	☐	☐	☐
b.Wear a helmet when riding a motorcycle, scooter	☐	☐	☐	☐
c. Drive the posted speed limit	☐	☐	☐	☐
d.Eat at least 5 servings of fruits and vegetables each day	☐	☐	☐	☐
e.Eat fast food more than once a week	☐	☐	☐	☐
f. Exercise at a moderate pace at least 13 minutes per day 5 days per week	☐	☐	☐	☐
g.Smoking cigarettes	☐	☐	☐	☐
h.Chew tobacco	☐	☐	☐	☐
i. Are exposed to secondhand smoke in your home or work	☐	☐	☐	☐
j. Use illegal drugs	☐	☐	☐	☐
k.Wash hands with soap and water after using the restroom	☐	☐	☐	☐
l. Wash hands with soap and water before preparing or eating meals	☐	☐	☐	☐
m. Apply sunscreen before plan time outside	☐	☐	☐	☐
n.Get a flu shot each year	☐	☐	☐	☐
o.Practice safe sex use condom or other by your methods	☐	☐	☐	☐
p.Take vitamins pills or supplements daily	☐	☐	☐	☐
q.Get enough sleep each night 7 to 9 hours	☐	☐	☐	☐
r. Feel stressed out	☐	☐	☐	☐
s. Feel happy about your life	☐	☐	☐	☐
t. Feel lonely	☐	☐	☐	☐
u. Worry about losing a job	☐	☐	☐	☐
v.Feel safe in your community	☐	☐	☐	☐

37. Have YOU been diagnosed by a doctor with any of the following health problems or disease? If you have not been diagnosed by a doctor with any of these health problems or diseases, please check the "No" box. If you have, please check the "Yes" box and then check the boxes in the other columns regarding that problem or disease.

Health Problem or Disease	No	Yes	I see a doctor	I am taking medications or getting treatment	I feel the disease is well managed
a. Diabetes	☐	☐	☐	☐	☐
b. Stroke	☐	☐	☐	☐	☐
c. High blood pressure	☐	☐	☐	☐	☐
d. High cholesterol	☐	☐	☐	☐	☐
e. Heart disease	☐	☐	☐	☐	☐
f. Cancer	☐	☐	☐	☐	☐
g. Asthma	☐	☐	☐	☐	☐
h. Respiratory/ lung disease	☐	☐	☐	☐	☐
i. Kidney disease	☐	☐	☐	☐	☐
j. Obesity	☐	☐	☐	☐	☐
k. Liver disease	☐	☐	☐	☐	☐
l. Arthritis	☐	☐	☐	☐	☐
m. Migraine headaches	☐	☐	☐	☐	☐
n. Mental disorders	☐	☐	☐	☐	☐

Health Problem or Disease	No	Yes	I see a doctor	I am taking medications or getting treatment	I feel the disease is well managed
o. Hepatitis	☐	☐	☐	☐	☐
p. Tuberculosis	☐	☐	☐	☐	☐
q. Epilepsy	☐	☐	☐	☐	☐
r. Lupus	☐	☐	☐	☐	☐
s. Sickle cell anemia	☐	☐	☐	☐	☐
t. Glaucoma	☐	☐	☐	☐	☐
u. Gonorrhea	☐	☐	☐	☐	☐
v. HIV/AIDS	☐	☐	☐	☐	☐
w. Dental health problems	☐	☐	☐	☐	☐
x. Hearing disorders	☐	☐	☐	☐	☐
y. Eye disorders	☐	☐	☐	☐	☐
z. Memory loss	☐	☐	☐	☐	☐
aa. Sinus problems	☐	☐	☐	☐	☐

38.Check all the special needs children in your home have faced (Check all that apply)

a.	My children (child living in my hose) do not have any special needs	☐
b.	Attention deficit / hyperactivity disorder (AD/HD)	☐
c.	Autism	☐
d.	Blindness/ visual impairment	☐
e.	Cerebral palsy	☐
f.	Child who uses a wheelchair	☐
g.	Deaf / hearing loss	☐
h.	Developmental delay	☐
i.	Down syndrome	☐
j.	Emotional disturbance	☐
k.	Epilepsy / Seizure disorder	☐
l.	Intellectual disability (formerly mental retardation)	☐
m.	Learning disabilities	☐
n.	Speech and language impairment	☐
o.	Spina bifida	☐
p.	Traumatic brain injury	☐
q.	Other (please specify)	☐

39. In this section, choose how much of a problem you think each item listed below is for your community where you live. Do think it is a serious problem, moderate problem, not a problem or, are you not sure about the issue in your community?

	Serious Problem	Moderate Problem	Not a Problem	Not Sure
a. Alcohol /drug use	☐	☐	☐	☐
b. Ambulance ser ices	☐	☐	☐	☐
c. Asthma /respiratory disorders	☐	☐	☐	☐
d. Eating disorders	☐	☐	☐	☐
e. Cancer	☐	☐	☐	☐
f. Child care/Day Care (safe, affordable, available)	☐	☐	☐	☐
g. Child abuse	☐	☐	☐	☐
h. Clean water/water pollution	☐	☐	☐	☐
i. Crime	☐	☐	☐	☐
j. Domestic violence	☐	☐	☐	☐
k. School systems	☐	☐	☐	☐
l. Elder abuse	☐	☐	☐	☐
m. Elder day care (safe, affordable, available)	☐	☐	☐	☐
n. Firearms	☐	☐	☐	☐
o. Healthcare affordability	☐	☐	☐	☐
p. Health care availability	☐	☐	☐	☐
q. Heart disease	☐	☐	☐	☐
r. High blood pressure	☐	☐	☐	☐
s. Strokes	☐	☐	☐	☐
t. Highway safety	☐	☐	☐	☐
u. HIV/AIDS	☐	☐	☐	☐

	Serious Problem	Moderate Problem	Not a Problem	Not Sure
v. Infant health	☐	☐	☐	☐
w. Infectious disease	☐	☐	☐	☐
x. Job availability	☐	☐	☐	☐
y. Job security	☐	☐	☐	☐
z. Mental illness	☐	☐	☐	☐
aa. Nursing home care (safe, affordable, available)	☐	☐	☐	☐
bb. Overweight adults	☐	☐	☐	☐
cc. Overweight children	☐	☐	☐	☐
dd. Prenatal health	☐	☐	☐	☐
ee. Recreation opportunities	☐	☐	☐	☐
ff. Secondhand smoke	☐	☐	☐	☐
gg. School violence	☐	☐	☐	☐
hh. Services for disabled	☐	☐	☐	☐
ii. Sewage treatment	☐	☐	☐	☐
jj. Sexually transmitted infections	☐	☐	☐	☐
kk. Smokeless tobacco	☐	☐	☐	☐
ll. Smoking	☐	☐	☐	☐
mm. Suicide	☐	☐	☐	☐
ccc. Trash /solid waste management	☐	☐	☐	☐
ddd. Unemployment	☐	☐	☐	☐
