# Supplementary material for: The prevalence and predictors of previous prostate cancer screening among men attending primary healthcare centers in Riyadh, Saudi Arabia
Source: Ann Med. 2026 Mar 14;58(1):2628366. doi: 10.1080/07853890.2026.2628366 (PMC12990262; doi:10.1080/07853890.2026.2628366)
Supplement: IANN-2025-3933.R3 supp file legends.docx [file IANN_A_2628366_SM3667.docx]

**Supplementary files legend/title**

**Supplementary material- for review**

Supplementary file 1: Health Need Assessment English Questionnaire
